# Assessing Residency Applicants’ Communication and Professionalism: Standardized Video Interview Scores Compared to Faculty Gestalt

**DOI:** 10.5811/westjem.2018.10.39709

**Published:** 2018-11-13

**Authors:** Benjamin H. Schnapp, Daniel Ritter, Aaron S. Kraut, Sarah Fallon, Mary C. Westergaard

**Affiliations:** University of Wisconsin, BerbeeWalsh Department of Emergency Medicine, Madison, Wisconsin

## Abstract

**Introduction:**

The Association of American Medical Colleges has introduced the Standardized Video Interview (SVI) to assess the communication and professionalism skills of residency applicants to allow a more holistic view of applicants beyond academic performance. Initial data suggests scores are not correlated with academic performance and provide a new measure of applicant attributes. It is not currently known how the SVI compares to existing metrics for assessing communication and professionalism during the interview process.

**Methods:**

Applicants to the University of Wisconsin Emergency Medicine Residency program were invited and interviewed without use of the SVI scores or videos. All faculty interviewers were blinded to applicants’ SVI information and asked to rate each applicant on their communication and professionalism on a scale from 1–25 (faculty gestalt score), analogous to the 6–30 scoring used by the SVI. We transformed SVI scores to our 1–25 system (transformed SVI score) for ease of comparison and compared them to faculty gestalt scores as well as applicants’ overall score for all components of their interview day (interview score).

**Results:**

We collected data for 125 residency candidates. Each applicant received a faculty gestalt score from up to four faculty interviewers. There was no significant correlation of SVI scores with faculty gestalt scores (Spearman’s rank correlation coefficient [r_s_] (123)=0.09, p=0.30) and no correlation with the overall interview score (r_s_(123)=0.01, p=0.93). Faculty gestalt scores were correlated positively with interview scores (r_s_(123)=0.65, p<0.01).

**Conclusion:**

SVI scores show no significant correlation with faculty gestalt scores of communication and professionalism. This could relate to bias introduced by knowledge of an applicant’s academic performance, different types of questions being asked by faculty interviewers, or lack of uniform criteria by which faculty assess these competencies. Further research is needed to determine whether SVI scores or faculty gestalt correlate with performance during residency.

## INTRODUCTION

Emergency medicine (EM) residency programs receive hundreds of applications each year for just a handful of residency positions. Residency applicants to Accreditation Council for Graduate Medical Education (ACGME)-accredited EM residencies submitted an average of 48.2 applications each to programs in 2017, a 50% increase from 32.2 applications each five years ago.^1^ The Association of American Medical Colleges (AAMC) has noted this trend across specialties and has encouraged students to apply to fewer programs, citing diminishing returns with an increased number of applications.^2^ Residency programs continue to search for methods of managing this increased volume of applicants, from the Standardized Letter of Evaluation (SLOE) aimed at allowing programs to rank applicants more effectively^3^ to coordinated interview days by institutions in the same geographic area, allowing applicants to curb the significant costs associated with travel to an increasing number of programs.^4^

Starting in the 2017–2018 application cycle, the AAMC implemented the Standardized Video Interview (SVI) as a pilot component of the Electronic Residency Application Service (ERAS®) for all applicants to EM residency programs. The SVI is intended to provide program directors (PDs) with standardized, reliable, and comparable information about applicants’ interpersonal communication skills and professionalism, allowing residency programs an additional data point by which to sort applicants, with a secondary goal of boosting the applications of applicants who might not otherwise have been considered.^5^ In past years, these characteristics could only be rated during in-person interviews. Questions were reviewed by subject matter experts in EM and graduate medical education and linked to ACGME competencies to ensure maximum validity.^6^ Following the 2017 pilot, it was demonstrated that there was no correlation between the SVI score and United States Medical Licensing Examination Step 1 exam scores, validating one of the objectives of the SVI: that it measure characteristics separate from academic knowledge.^5^ It has yet to be determined, however, the level of correlation that exists between SVI score and how applicants are currently evaluated by faculty during in-person interviews. The goal of this study was to determine the correlation of the AAMC’s SVI score to interview faculty gestalt of professionalism and communication skills during in-person interviews at the University of Wisconsin Emergency Medicine Residency program during the 2017–2018 application cycle. Our hypothesis was that the SVI scores should correlate with our faculty’s gestalt of communication skills and professionalism.

## METHODS

### SVI

When taking the SVI, the applicant receives a total of six questions, one at a time, presented on a personal computer. They are allowed up to 30 seconds to prepare their answer, and then up to three minutes to record their response. Questions are not provided prior to the start of the interview. Questions were first vetted by a group of residency PDs for potential bias and relevance to ACGME competencies.^7^ Each of the applicant’s answers are then graded by six different trained evaluators who use a standardized rating scale developed by PDs and have an opportunity to practice and receive feedback using the rating scale. Raters also receive training in unconscious bias. Each question is rated on a five-point scale with total scores falling between 6–30 (pilot mean: 19.1, standard deviation [SD] 3.1). Applicants are unable to retake the SVI or void their score. An applicant’s video and score are available for viewing by EM residency program leadership within the applicant’s ERAS application during the current pilot.

**Population Health Research Capsule**

What do we already know about this issue?

*Communication and professionalism are essential aspects of competent physicians and could previously only be evaluated by residencies during the in-person interview. The Association of American Medical Colleges (AAMC) introduced the Standardized Video Interview (SVI) to assess applicants’ competency in these domains*.

What was the research question?

How do SVI scores compare to faculty evaluations of applicants’ communication and professionalism?

What was the major finding of the study?

*There was no correlation between SVI scores and faculty communication and professionalism scores*.

How does this improve population health?

*SVI scores appear to provide novel data to the residency application process which could aid in the selection of physicians with strong professionalism and communication skills. More research is needed to determine how SVI scores correlate with residency performance*.

### Setting

The University of Wisconsin Emergency Medicine Residency is a three-year residency program with 12 residents per year, based at a tertiary-care hospital located in Madison, Wisconsin.

### Applicant Screening

During the applicant screening process, the program director (PD) and the two assistant program directors (APD) reviewed each applicant’s entire file with the exception of the SVI information, which was not examined in any way. Applicants were assigned a composite score based on academic and clinical achievement, which was used to generate a list of applicants invited to interview. The screening and invitation process was identical to what had been used in prior years before the SVI information was available.

### Applicants

All applicants who attended an interview at the University of Wisconsin Emergency Medicine Residency program during the 2017–2018 interview season were eligible for inclusion; there were 11 interview days in total. We excluded internal applicants (from the University of Wisconsin School of Medicine and Public Health) since faculty may have had previously formed opinions about their communication skills and professionalism.

### Interview Day

Prior to each interview day, all faculty members who were interviewing applicants received the ERAS file for each applicant. At the start of each day, one of the investigators gave faculty a standard set of instructions. Interviewers were asked to conduct their interview in their usual fashion (unstructured—ranging from casual conversation to behavioral interview questions depending on individual faculty preference), but to use applicants’ responses to these questions to rate their competence in the areas of communication and professionalism alone, with a score of 1 representing the least effective professionalism and communication skills an applicant could demonstrate and a 25 representing the most advanced professionalism and communication skills an applicant could demonstrate. This score was dubbed the “faculty gestalt score.” A 1–25 scale was chosen to mirror the SVI’s 6–30 scoring scale as closely as possible, removing the additional complexity of a nonstandard starting integer. While the SVI scores applicants from 1–5 across six individual domains to generate a composite final score, this was deemed unfeasible for faculty to complete for each applicant during the brief duration of a standard residency interview; hence, a single gestalt score was used instead.

Immediately after each interview, each interviewer recorded the score for the applicant’s communication and professionalism based on the interview alone along with their overall interview day score for each applicant (the “interview score”), which was based on a much broader range of factors (e.g., personal statement, research experience, academic interests) and was identical to the system used in previous years to evaluate and rank applicants.

Every applicant interviewed with four faculty members during their interview day: the PD and one of the APDs interviewed all applicants, while the other APD was present for every interview day but did not interview every applicant, and the remainder of the interviews were performed by other members of the faculty group. A total of 19 different faculty members participated in the interview season. All interviewers were core faculty members who use the ACGME EM Milestones to help assess residents after each shift, including the Interpersonal and Communication Skills (ICS) and Professionalism (PROF) milestones.

After each interview day, the professionalism and communication faculty gestalt scores were recorded by the residency coordinators in a confidential, secure spreadsheet along with the overall interview scores, which only the residency leadership team had access to. Other than the addition of the professionalism and communication faculty gestalt score, the process was identical to the previous year’s interview process for applicants.

### Analysis

All of the SVI scores for applicants were transformed to our 1–25 rating scale by subtracting five from each score for ease of comparison and interpretation. This new score became the “transformed SVI score.” With an alpha = .05 and power = 0.80, the projected sample size needed to detect a medium correlation (r_s_=0.30) is approximately n = 85. For each applicant, a mean of all available faculty gestalt scores was calculated. We used Microsoft Excel to compute ranges, medians and means and SPSS was used to calculate Spearman’s rank correlation coefficient (r_s)_ for the transformed SVI score, the professionalism and communication faculty gestalt score, and the overall interview score. Krippendorf’s alpha (similar to Cohen’s kappa with similar standards for acceptable agreement^8^ but allows for missing data) for faculty gestalt scores was calculated using ReCal OIR, available at http://dfreelon.org/utils/recalfront/recal-oir/.

This study was deemed to be exempt from full institutional review board (IRB) review by the The University of Wisconsin Health Sciences IRB. This study also received approval from the AAMC to use SVI pilot data.

## RESULTS

A total of 125 applicants were included in the analysis, with 423 faculty gestalt scores total out of a possible 500 generated over the interview season. Means and SDs for the transformed SVI score and the faculty gestalt score are listed below in the Table. The mean transformed SVI score was 14.6 (+/− 2.6), the mean faculty gestalt score was 17.9 (+/− 3.0), and the mean interview score was 6.7 (+/− 1.7).

**Figure 1:**
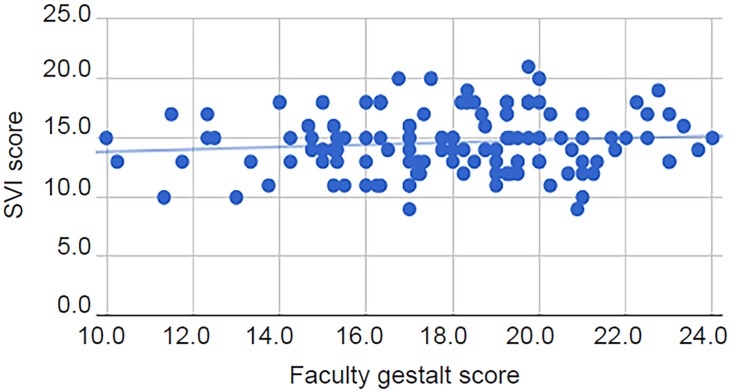
Scatter plot of transformed Standardized Video Interview (SVI) score vs. faculty gestalt score (r_s_(123)=0.09, p=0.30), with line of best fit.

**Figure 2:**
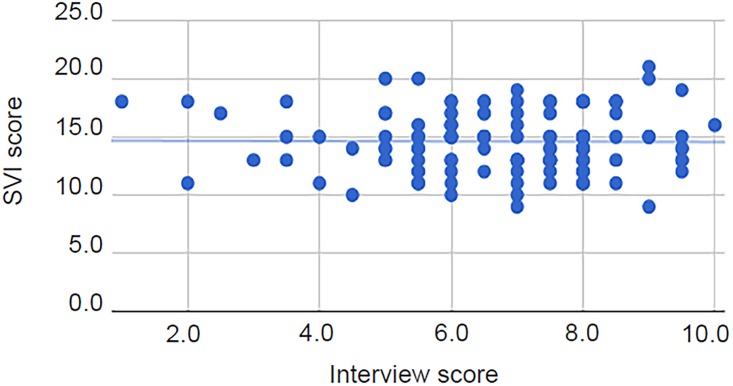
Scatter plot of transformed Standardized Video Interview (SVI) score vs. interview score (r_s_(123)=0.01, p=0.93), with line of best fit.

**Figure 3:**
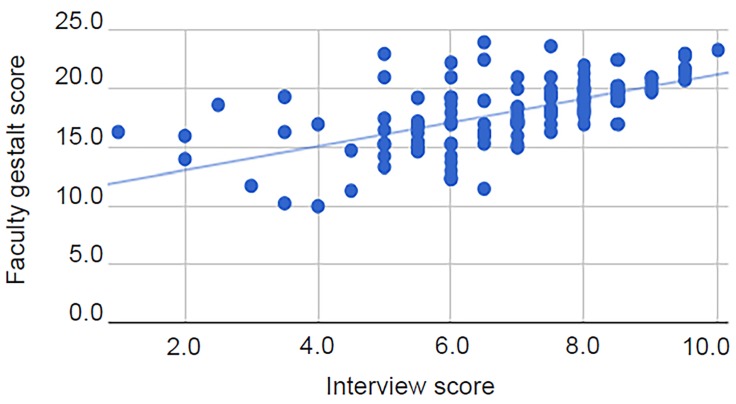
Scatter plot of faculty gestalt score vs. interview score (r_s_(123)=0.65, p<0.01), with line of best fit.

There was no significant correlation (r_s_(123)=0.09, p=0.30) between transformed SVI scores and faculty gestalt scores (Figure 1). Additionally, there was no correlation (r_s_(123)=0.01, p=0.93) between overall interview scores and transformed SVI scores (Figure 2). There was, however, a significant correlation with a medium effect (r_s_(123)=0.65, p<0.01) between the faculty gestalt score and the overall interview score (Figure 3).

We calculated Krippendorff’s alpha to determine the agreement between faculty raters of communication and professionalism and found it to be 0.26, suggesting low inter-rater reliability.

## DISCUSSION

Our results showed no significant correlation between SVI scores and faculty gestalt. For our residency program, the SVI appears to provide new and unique applicant data that differs from any data currently generated during the interview process. The challenge for programs centers on whether and how to use this information during the recruitment season.

According to the AAMC, EM programs should consider adding the SVI score to the composite score during the initial applicant screening process to determine which applicants are invited to interview.^9^ This stage of the application process generally includes data such as USMLE step scores and SLOE performance. Incorporation of the SVI at this early stage would allow for consideration of non-academic factors into the screening process; however, it could also give it an impact beyond that of many other available ERAS data points not frequently used in the composite score, such as leadership or research experience. Determining the ideal weight of the SVI score at this early stage compared with other composite score elements poses a particular challenge when the link between SVI scores and residency performance has not been firmly established.^7^ Our research further suggests that using SVI scores during this stage as a surrogate for how faculty would feel about an applicant’s professionalism and communication would not be effective, since the two scores were not correlated.

It is not surprising that faculty gestalt and overall interview scores are highly correlated, as they are based on observation of the same interview. One potential explanation for the disparity between SVI and faculty gestalt scores, however, lies in what is being assessed by faculty gestalt. While faculty were instructed to score professionalism and communication skills as objectively as possible when generating a faculty gestalt score, an applicant’s “fit” or similarity to existing residents and faculty can color assessments when evaluating potential trainees and future colleagues. This sense of how an applicant will fit within the culture of a program and institution inevitably invokes implicit bias, which can have significant long-term consequences for diversity, inclusion, and overall program identity. It can also vary greatly between individuals, as seen in the low inter-rater reliability of our faculty gestalt scores, consistent with prior literature on the unreliability of unstructured interviews.^10^ Despite knowledge of this variability, residencies continue to put great faith in interviewers’ aggregate impression of applicants’ skills and potential for success.^11^ Perhaps, then, an objective SVI score is a welcome addition to our interview process, introducing a more objective measure of interpersonal skills than has previously been possible.

Alternatively, a more concerning possibility raised by this study is that the SVI score does not measure the professionalism and communication skills that it purports to measure, instead measuring other variables such as applicants’ performance and improvisation skills, an objection that has been raised before.^12^ Accurately assessing interpersonal skills via a one-way interaction with technology may be inherently problematic and does not have the same base of validity evidence for assessing resident competency in the literature as techniques such as the Mini-Clinical Evaluation Exercise and 360-degree evaluations.^13^ Similarly, while professionalism assessments around ethics and moral reasoning (such as the SVI) have been shown to be reliable and valid,^14^ there is concern that these may not translate well to observed behaviors.^15^ Finally, while the SVI currently does not cost students money during the pilot phase,^5^ the significant resources required to execute this project suggest that it is unlikely to remain free to students. As the cost of applying and interviewing for residency is already estimated at ≈ $8,000 per student,^16^ there should be significant caution with burdening students with a further source of stress and cost when the value it provides is currently unclear.

## LIMITATIONS

Our study has several important limitations. The sample size was small, and it was drawn from a single year of data collected at one institution. Some of the faculty gestalt scores were missing, likely due to interviewers forgetting to record a score or running out of time. However, missing scores represented a relatively small portion of the data. To create similarity with the SVI’s 6–30 scoring, we used a 1–25 scoring system; however, it may have been difficult for faculty to differentiate between scores with this many items in the rating scale (e.g., between a 20 and a 21).

Although interviewers received standardized instruction at the beginning of each interview day, we did not formally train them, provide formalized feedback, or define how they should rate an applicant’s professionalism and communication, raising the possibility that interviewers assessed and rated these qualities differently. Interviewers also had access to applicant’s application files when assigning faculty gestalt scores, raising the possibility that factors other than interview performance affected their scores. While our core faculty are familiar with the EM Milestones and use them frequently to assess residents, we did not specifically review the content of the interpersonal communication skills and Professionalism milestones with faculty as a part of this study. Interviewer implicit bias is an additional concern that was not assessed by this study. We believe that overall, however, our methods are likely representative of how other institutions currently assess applicant communication and professionalism skills during the interview process.

## CONCLUSION

SVI scores for our cohort of 2017–2018 applicants showed no correlation with our faculty’s gestalt rating of applicants’ communication and professionalism during their interviews. It is unclear at this time if either metric is correlated with future applicant success. The uncertainty about the current value of the SVI score represents an opportunity for future research prior to a broader roll-out, which might explore the potential correlations between SVI scores and success in the match, professionalism citations, clinical performance, and Press-Ganey ratings.

## Figures and Tables

**Table:** Range, median and mean transformed Standardized Video Interview (SVI), faculty gestalt, and interview scores, with standard deviations (SD).

	Transformed SVI score	Faculty gestalt score	Overall interview Score
Range	9–21	2–25	1–10
Median	15	19	7
Mean	14.6	17.9	6.7
SD	2.6	3.0	1.7

## References

[1] Association of American Medical Colleges (2017). Emergency Medicine ACGME Residency Match Data By Applicant. ERAS Statistics: Association of American Medical Colleges.

[2] Mann S (2017). How Many Residency Applications?. AAMC News.

[3] Girzadas DV, Harwood RC, Dearie J (1998). A comparison of standardized and narrative letters of recommendation. Acad Emerg Med.

[4] Shappell E, Fant A, Schnapp B (2017). A novel collaboration to reduce the travel-related cost of residency interviewing. West J Emerg Med.

[5] (2018). SVI FAQ.

[6] Bird S, Blomkalns A, Deiorio NM (2018). Stepping up to the plate: emergency medicine takes a swing at enhancing the residency selection process. AEM Educ Train.

[7] (2017). AAMC Standardized Video Interview Update.

[8] Kirppendorff K (1989). Content Analysis: An Introduction to Its Methodology.

[9] Association of American Medical Colleges (2017). Using AAMC Standardized Video Interview Scores in Residency Selection: A Resource Guide.

[10] Dana J, Dawes R, Peterson N (2013). Belief in the unstructured interview: the persistence of an illusion. Judgm Decis Mak.

[11] Crane JT, Ferraro CM (2000). Selection criteria for emergency medicine residency applicants. Acad Emerg Med.

[12] Buckley RJ, Hoch VC, Huang RD (2018). Lights, camera, empathy: a request to slow the Emergency Medicine Standardized Video Interview Project Study. AEM Educ Train.

[13] Chan TM, Wallner C, Swoboda TK (2012). Assessing interpersonal and communication skills in emergency medicine. Acad Emerg Med.

[14] Rodriguez E, Siegelman J, Leone K (2012). Assessing professionalism: summary of the working group on assessment of observable learner performance. Acad Emerg Med.

[15] Tiffin PA, Finn GM, McLachlan JC (2011). Evaluating professionalism in medical undergraduates using selected response questions: findings from an item response modelling study. BMC Med Educ.

[16] Blackshaw AM, Watson SC, Bush JS (2017). The cost and burden of the residency match in emergency medicine. West J Emerg Med.

